# PREDICTION OF LONG-TERM FUNCTIONAL OUTCOME FOLLOWING DIFFERENT REHABILITATION PATHWAYS AFTER STROKE UNIT DISCHARGE

**DOI:** 10.2340/jrm.v56.19458

**Published:** 2024-05-21

**Authors:** Malin C. NYLÉN, Tamar ABZHANDADZE, Hanna C. PERSSON, Katharina S. SUNNERHAGEN

**Affiliations:** 1 Institute of Neuroscience and Physiology, Rehabilitation Medicine, University of Gothenburg, Gothenburg; 2 Department of Occupational Therapy and Physiotherapy, Sahlgrenska University Hospital, Gothenburg; 3 Neurocare, Sahlgrenska University Hospital, Gothenburg, Sweden

**Keywords:** stroke, rehabilitation, functional outcome, activities of daily living, independence

## Abstract

**Objective:**

To investigate whether referral for different types of rehabilitation on discharge from Swedish stroke units can predict functional outcomes at 1 and 5 years after a stroke.

**Design:**

A longitudinal and registry-based study.

**Subjects/patients:**

A total of 5,118 participants with index stroke in 2011 were followed-up at 1 and 5 years after the stroke.

**Methods:**

Ordinal logistic regression models were developed to predict the category of functional outcome: independent, dependent, or dead. The primary predictors were planned rehabilitation in a home setting, inpatient rehabilitation, and outpatient rehabilitation, with no planned rehabilitation as the reference category.

**Results:**

Planned outpatient rehabilitation predicted independence (compared with death) at 1 year. Planned rehabilitation in the home setting predicted independence (compared with death) at 1 and 5 years. Compared with other planned pathways, participants planned for inpatient rehabilitation had more severe conditions, and planned inpatient rehabilitation did not predict independence.

**Conclusion:**

Planning for outpatient or home-based rehabilitation appeared to lead more effectively to participants achieving independence over the course of 1–5 years. This may have been due to the less severe nature of these participants’ conditions, compared with those requiring inpatient rehabilitation.

LAY ABSTRACT

Patients discharged from stroke units who require rehabilitation may receive training provided in specialized facilities, clinics, or through home-based programmes. Here we investigated whether these different rehabilitation pathways could predict patients’ functional levels at 1 and 5 years post-stroke. We analysed data from 5,118 participants, and found that patients planned for home-based rehabilitation were more likely to survive and to manage daily activities, either independently or with assistance, compared with those without this kind of rehabilitation plan. In contrast, patients with planned inpatient rehabilitation were less likely to be alive or independent at follow-up. Our results suggest that different rehabilitation pathways have different effects on recovery. Notably, the patients referred to these different forms of rehabilitation also possess diverse characteristics, which must be considered when evaluating the effectiveness of each rehabilitation pathway, as patient characteristics also influence the observed outcomes.

In comprehensive stroke units, medical surveillance and rehabilitation improve survival and functional outcome after stroke (1). Rehabilitation needs should be identified before discharge from a stroke unit. Once these needs are assessed, rehabilitation can then be organized into one of three pathways: inpatient, outpatient, or home-setting rehabilitation. In Sweden, these forms of rehabilitation are funded with public taxes. Specialized inpatient rehabilitation is provided by some hospitals in Sweden, and is primarily available to patients with severe strokes and complex needs. Patients referred for inpatient rehabilitation are typically transferred to these rehabilitation units within a month after stroke onset (2); however, the settings, rehabilitation programmes, and facilities can differ between hospitals. Outpatient rehabilitation can be organized at primary care or rehabilitation clinics, and it is a suitable option for patients who are medically stable and have less complex needs. Patients with mild-to-moderate stroke can be offered early supported discharge services (3), which are organized by a multidisciplinary team at the stroke unit and serve as a continuation of stroke unit rehabilitation after discharge. For patients who are not eligible for early supported discharge, but are discharged home, local municipalities can offer home-based rehabilitation (4).

Diabetes mellitus, atrial fibrillation, stroke recurrence, and severe stroke are factors associated with reduced long-term survival after stroke (5, 6), while independent activities of daily living before stroke and younger age are associated with lower risk of death or dependence at 5 years after stroke (7). Over the past 15 years, a temporal change towards reduced stroke case fatality has been observed, and increased proportions of patients have been discharged to home and to rehabilitation (8). This suggests that improvements in stroke care and rehabilitation have yielded better functional outcomes. Notably, limited information is available regarding how different rehabilitation path-ways may influence long-term functional outcomes. It can be assumed that patient characteristics such as comorbidities, age, and stroke type and severity interfere with the results from rehabilitation. However, the importance of these factors when planning rehabilitation is unknown. Bridging these knowledge gaps in the existing literature could potentially lead to more specific and individualized strategies for planning rehabilitation after stroke, with consideration of relevant patient characteristics.

In the present study, our primary aim was to investigate whether referral for different types of rehabilitation on discharge from Swedish stroke units could predict functional outcomes at 1 and 5 years after a stroke. The secondary aim was to investigate changes in functional outcomes between these time-points.

## METHODS

### Data availability

The data supporting the findings of this study cannot be made publicly available or shared due to Swedish regulations. Qualified researchers in relevant fields may request access to the dataset from the registry holder, Riksstroke, by email: riksstroke@regionvasterbotten.se.

### Study sample and procedure

This registry-based longitudinal study included 5,118 participants. The findings are reported in accordance with the Strengthening the Reporting of Observational Studies in Epidemiology (STROBE) guidelines for cohort studies (9). Data were extracted from the Swedish national register Riksstroke, which includes 90% national coverage of stroke occasions and contributions from all 72 acute care hospitals in Sweden. We used the Riksstroke acute module and 1- and 5-year follow-up data (10). All baseline data were obtained from Riksstroke’s acute module, recorded by nurses at stroke units. Data regarding patient health status and functional levels before stroke, and data related to acute hospitalization were transferred from each hospital to Riksstroke, as organized by registered nurses and statisticians at Riksstroke. Riksstroke conducted a follow-up survey of all registered participants after 1 year following stroke. After 5 years, Riksstroke randomly selected 50% of the participants who were registered in the acute module with index strokes in 2011, to be invited to participate in a survey. The surveys were completed by either the participants, their next of kin, or healthcare professionals. At both 1 and 5 years, the follow-up surveys were distributed by mail, with 2 reminders sent for each follow-up.

**Fig. 1:**
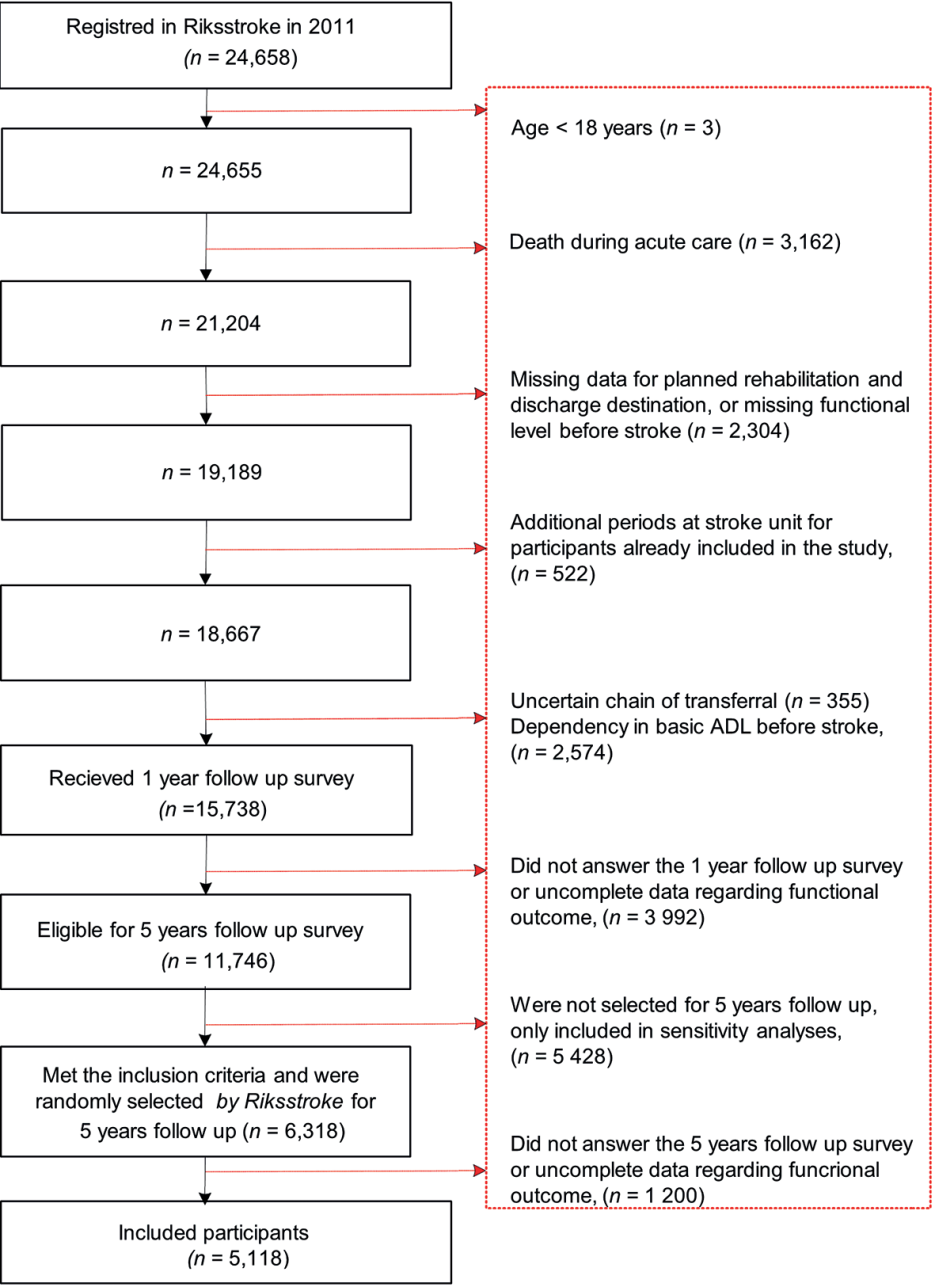
Inclusion of study participants. ADL: activities of daily living.

If a participant received treatment at a stroke unit more than once in 2011, data from the first period were used. If a second treatment period occurred < 4 days after discharge, the two periods were considered a single period (Fig. 1). Individuals were included in the present study if they were diagnosed with stroke in 2011 according to the International Classification of Diseases, aged ≥ 18 years, independent in basic activities of daily living before stroke, and selected for the 5-year follow-up survey (Fig. 1). Individuals were excluded if they died during the hospital stay, were missing data concerning planned rehabilitation after discharge from acute stroke care, had an unclear chain of transfer, or were missing data regarding the outcome variable.

### Definitions and measurements

Outcome variables were derived from Riksstroke’s 1- and 5-year follow-up surveys. Functional outcomes at 1 and 5 years post-stroke were categorized into 3 groups: deceased (those who passed away within 1 year post-stroke, or between 1 and 5 years after the stroke), dependent (requiring help with mobility, toilet use, or dressing), and independent (not requiring help with mobility, toilet use, or dressing) (11).

The independent variable of main interest was planned rehabilitation, defined as the rehabilitation planned at discharge from the stroke unit. Participants could be planned for rehabilitation in a home setting (managed by a stroke unit or provided by a municipality), inpatient rehabilitation (stroke-specific rehabilitation at a hospital), or outpatient rehabilitation (at primary care or outpatient clinics). Participants not planned to receive any of these types of rehabilitation were classified as no planned rehabilitation.

Stroke type was defined as an ischaemic stroke or haemorrhage. Stroke severity was determined based on the level of consciousness at hospital admission, categorized as follows: alert on hospital arrival or affected consciousness (drowsy or unconscious). Reperfusion treatment was defined as having undergone thrombolysis, thrombectomy, or both. Atrial fibrillation and diabetes were recorded if diagnoses were confirmed during acute hospitalization or from a previous investigation.

### Statistical analyses

Participants’ descriptive statistics are presented for the total sample, as well as stratified according to inpatient, outpatient, or home-based rehabilitation, or no planned rehabilitation. Functional outcomes after 1 and 5 years were recorded for each planned rehabilitation group. Between-group differences for the planned rehabilitation types, as well as for included versus excluded participants, were calculated using the χ^2^ test for categorical variables, or the Mann‒Whitney *U* test for continuous variables. For each group, we calculated the proportional change in the number of participants reporting independence after 1 and 5 years. We used a discrete-time Markov chain model to analyse the probabilities of transitions between conditions from 1 year to 5 years, with conditions defined as independent, dependent, or dead. Probabilities were calculated for each possible transition from one state to another, from year 1 to year 5 (4-year interval).

Ordinal logistic regression was used to identify factors predicting functional outcomes at 1 and 5 years after stroke. The outcomes were ordinal variables with 3 response levels: independent, dependent, or dead. Eligible covariates were selected based on clinical knowledge and previous research (12–15), and their potential interactions were examined by plotting a directed acyclic graph (16). The covariates were checked for multicollinearity, and a high correlation was defined as an absolute value of > 0.7. The primary independent variables were home-setting rehabilitation, outpatient rehabilitation, and inpatient rehabilitation, with a reference category of no rehabilitation. The covariates were age (continuous variable), sex (female or male [reference category]), stroke type (ischaemic or haemorrhagic [reference category]), consciousness at hospital arrival (alert or drowsy/unconscious [reference category]), previous stroke (yes or no [reference category]), atrial fibrillation (yes or no [reference category]), and diabetes (yes or no [reference category]).

Individual models were fitted to predict functional outcomes after 1 and 5 years. The ordinal regression analyses included data from 5,025 participants. This data set was randomly divided into a training dataset (80%, *n* = 4,021) used to fit the models, and a test (20%, *n* = 1,004) dataset used to evaluate the models’ predictive performance. Classification tables were created for the training and test datasets. We fitted partial proportional ordinal regression models, using a statistical technique for modelling ordinal response variables that enables the proportional odds assumption to be relaxed for some predictor variables. This method allows for more flexibility compared with traditional ordinal regression models, by permitting certain covariates to have varying effects across different thresholds of the ordinal outcome, i.e. where all levels of the ordinal dependent variable do not necessarily have a consistent predictive value for the outcome. R^2^ was determined to evaluate the proportion of variation in the dependent variable that could be explained by the independent variables. The Hosmer–Lemeshow test was used to evaluate the goodness of fit, with a non-significant *p*-value indicating a good fit (17). The C-index was used to evaluate model performance, with a C-index of 0.5 indicating no discriminatory ability, and a C-index of 1.0 indicating perfect discriminatory ability (18). SAS 9.4 (SAS Institute, Cary, NC, USA) was used for ordinal logistic regressions. The level of significance was set at α = 5%. Transition probabilities were calculated using the package Markov chain (19) in R statistics 4.2.1 (R Foundation for Statistical Computing, Vienna, Austria).

## RESULTS

**Fig. 2:**
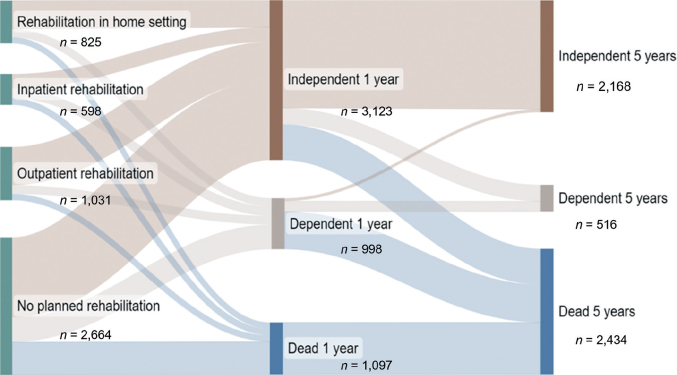
Sankey diagram showing functional outcomes after 1 and 5 years, for participants with different types of planned rehabilitation after discharge from a stroke unit (*n* = 5,118).

We observed significant differences in characteristics between the included participants (*n* = 5,118) and excluded individuals (*n* = 19,450). Compared with the excluded individuals, the included participants had a lower median age, higher proportion of men, and higher proportion of participants who were conscious upon hospital arrival (*p* < 0.0001). The study sample comprised 5,118 participants (Table I). The median age at stroke onset was 76 years (interquartile range, 16 years), 56% were men, and 90.1% were alert upon hospital admission (Table I). Compared with the other participant groups, the outpatient rehabilitation group had a higher proportion of men, and younger participants (median age, 72 years) (*p* < 0.0001) (Table I). Planning for inpatient rehabilitation was more common among participants with haemorrhage than those with ischaemic stroke (Table I). Participants who were planned for inpatient rehabilitation had a shorter median stay at the stroke unit (6 days), compared with those in other groups (*p* < 0.0001). Being alert upon hospital arrival was less common among participants planned for inpatient rehabilitation compared with those in the other groups (*p* < 0.0001). Participants without planned rehabilitation were older (median age, 78 years) than those with planned rehabilitation (*p* < 0.0001). The group without planned rehabilitation included greater proportions of women (*p* = 0.005) and participants with history of previous stroke (*p* = 0.02) (Table I). At the 1-year follow-up, 3,123 participants were independent, with or without planned rehabilitation. However, by the 5-year follow-up, this number decreased by 31%, resulting in 2,168 participants maintaining their independence. This decrease in independent participants between 1 and 5 years was 42% among those planned for inpatient rehabilitation, and 27% among those planned for outpatient rehabilitation (Fig. 2, Table II).

**Table I:** Characteristics of study participants: total sample, and stratified based on planned rehabilitation after discharge from stroke unit

	All participants (*n* = 5,118)	Planned rehabilitation after discharge from stroke unit^a^			
		No planned rehabilitation (*n* = 2,664)	Home setting (*n* = 825)	Inpatient rehabilitation (*n* = 598)	Outpatient rehabilitation (*n* = 1,031)
Age, years, mean (SD)	75.2 (11.4)	76.3 (11.3)	75 (10.9)	76.0 (11.8)	71.8 (11.1)
Median (IQR)	76 (16)	78 (16)	77 (15)	78 (17)	72 (15)
Sex, *n* (%)					
Men	2,846 (55.6)	1,432 (53.7)	451 (54.7)	319 (53.3)	644 (62.5)
Women	2,272 (44.4)	1,232 (46.3)	374 (45.3)	279 (46.7)	387 (37.5)
Stroke type, *n* (%)					
Haemorrhage	468 (9.1)	213 (8.0)	65 (7.9)	80 (13.4)	110 (10.7)
Ischaemic stroke	4,650 (90.9)	2,451 (92)	760 (92.1)	518 (86.6)	921 (89.3)
Level of consciousness, *n* (%)^b^					
Alert on arrival at hospital	4,614 (90.1)	2,393 (89.8)	774 (93.4)	502 (83.95)	945 (91.7)
Drowsy on arrival at hospital	379 (7.4)	198 (7.4)	36 (4.4)	76 (12.7)	69 (6.7)
Unconscious on arrival at hospital	72 (1.4)	45 (1.7)	9 (1.1)	10 (1.7)	8 (0.8)
Reperfusion treatment, *n* (%)	356 (7.0)	160 (6.0)	68 (8.2)	44 (7.4)	84 (8.2)
Previous stroke, *n* (%)^c^	901 (17.7)	499 (18.8)	128 (15.6)	100 (16.9)	174 (16.9)
Atrial fibrillation, *n* (%)^d^	1,399 (27.5)	758 (28.6)	217 (26.5)	184 (30.1)	240 (23.4)
Medical treatment for hyper-tension, *n* (%)^e^	3,137 (61.6)	1,655 (62.4)	499 (60.6)	369 (62.1)	614 (59.8)
Diabetes, *n* (%)^f^	957 (18.8)	488 (18.37)	147 (17.9)	121 (20.3)	201 (19.5)
Smoking, *n* (%)^g^	599 (12.4)	288 (11.6)	95 (12.1)	74 (13.4)	142 (14.3)
Length of stay at stroke unit^h^					
Mean (SD), days	12.2 (15.1)	11.6 (14.9)	11.9 (13.5)	9.8 (12.5)	15.2 (17.4)
Median (IQR), days	7 (11)	6 (12)	7 (11)	6 (8)	10 (15)

a Planned rehabilitation was defined as the rehabilitation planned for the patient upon discharge from the stroke unit. Missing data *n* (%):

b 53 (1),

c 24 (0.5),

d 28 (0.5),

e 23 (0.5),

f 14 (0.3),

g 299 (5.8),

h 196 (3.8).

IQR: interquartile range; SD: standard deviation.

**Table II:** Functional outcome stratified according to planned rehabilitation after discharge from stroke unit (*n* = 5,118)

Outcome	Independent	Dependent	Dead
1-year outcome			
No planned rehabilitation from stroke unit	1,550 (58.2)	479 (18.0)	635 (23.4)
Rehabilitation in a home setting, including ESD	545 (66.1)	160 (19.4)	120 (14.6)
Inpatient rehabilitation	286 (47.8)	183 (30.6)	129 (21.6)
Outpatient rehabilitation	742 (72.0)	166 (16.1)	123 (11.9)
5-year outcome			
No planned rehabilitation from stroke unit	1,085 (40.7)	214 (8.0)	1,365 (51.2)
Rehabilitation in a home setting, including ESD	375 (45.5)	98 (11.9)	352 (42.7)
Inpatient rehabilitation	165 (27.6)	86 (14.4)	347 (58)
Outpatient rehabilitation	543 (52.7)	118 (11.5)	370 (35.9)

ESD: early supported discharge.

**Fig. 3:**
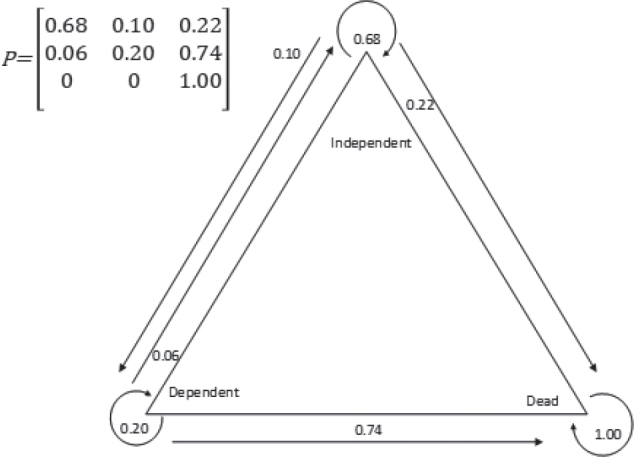
A discrete time Markov chain presenting the probabilities of transition between states from 1 year after stroke to 5 years after stroke.

The Markov model, developed from the probabilities of transitions between participants’ condition between the two follow-ups (Fig. 3), included one end state: death. Among participants who were independent at the 1-year follow-up, the probability of staying independent after 5 years was 68%. There was a 10% probability of transition from independence to dependence, and a 22% probability of transition from independence to death. Among participants who were dependent at the 1-year follow-up, there was a 20% probability of still being dependent after 5 years, a 6% probability of transition to independence, and a 74% probability of transition to death (Fig. 3).

**Fig. 4:**
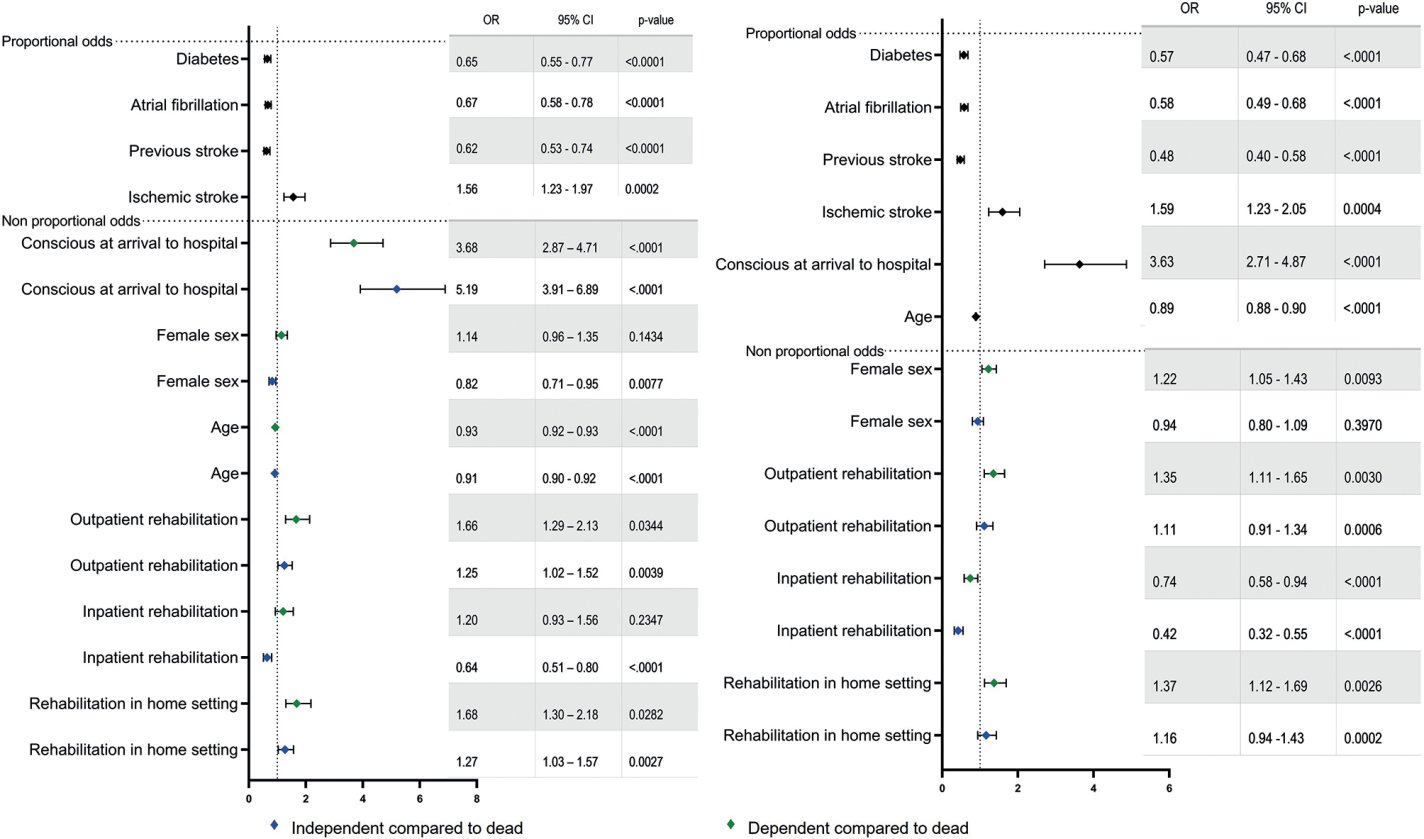
(A) Results of the partial proportional ordinal regression model used to identify factors explaining outcome at 1 year following stroke. (B) Results of the partial proportional ordinal regression model used to predict functional outcome at 5 years after stroke. OR: odds ratio, CI: confidence interval.

Planned home-based rehabilitation was associated with higher odds of independence (OR: 1.27, 95% CI: 1.03–1.57) and dependence (OR: 1.68, 95% CI: 1.30–2.18) compared with death at 1 year after stroke (Fig. 4). Participants with planned inpatient rehabilitation had lower odds of independence compared with death at the 1-year follow-up (OR: 0.64, 95% CI: 0.51‒0.80). Planned outpatient rehabilitation was associated with higher odds of independence (OR: 1.25, 95% CI: 1.02–1.52) and dependence (OR: 1.66, 95% CI: 1.29–2.13) compared with death at 1 year post-stroke (Fig. 4). The model was developed using the training dataset (*n =* 4,018, R^2^: 0.26, C-index: 0.77, Hosmer‒Lemeshow goodness-of-fit test: *p* = 0.17). Data from the remaining participants were used to evaluate the predictive performance of the test dataset (*n* = 1,004; R^2^: 0.27). Classification tables for the training and test datasets are available in Tables SI and SII.

Planned rehabilitation in a home setting was associated with higher odds of independence (OR: 1.16; 95% CI: 0.94–1.43) and dependence (OR: 1.37; 95% CI: 1.12–1.69) compared with death at the 5-year follow-up (Fig. 4). Participants planned for inpatient rehabilitation had lower odds of independence (OR: 0.42; 95% CI: 0.32‒0.55) and dependence (OR: 0.74; 95% CI: 0.58‒0.94) compared with death at 5 years after stroke. Participants planned for outpatient rehabilitation had higher odds of independence (OR: 1.11; 95% CI: 0.91‒1.34) and dependence (OR: 1.35; 95% CI: 1.11‒1.65) compared with death at the 5-year follow-up (Fig. 4). The model was developed using the training dataset (*n* = 4,018; R2: 0.34, C-index: 0.81, Hosmer‒Lemeshow goodness-of-fit test: *p* = 0.002). Data from the remaining participants were used to evaluate the predictive performance of the test dataset (*n* = 1,004; R2: 0.36). Classification tables for training and test datasets are available in Tables SIII and SIV.

## DISCUSSION

Our present analysis of a national clinical stroke cohort revealed that participants who were planned for home-based rehabilitation and outpatient rehabilitation had higher odds of being independent at the 1-year and 5-year follow-ups, compared with participants planned for inpatient rehabilitation. Participants with planned rehabilitation in a home setting had the highest odds of being independent or dependent, compared with dead, with adjustments for age, sex, comorbidities, stroke type, and stroke severity. These participants were eligible for discharge to their home, and previous trials have confirmed beneficial functional outcomes for people with stroke who receive rehabilitation in a home setting (3). Participants who were planned for outpatient rehabilitation had high odds of independence, compared with dependence and death, at 1 year and 5 years after stroke. Participants in this group were considered likely to be able to commute to the rehabilitation centre, which implies a certain level of function. Our findings suggested substantially better outcomes for participants planned for outpatient rehabilitation and home-based rehabilitation, suggesting that these targeted rehabilitation pathways may contribute to patient independence.

Participants who were planned for inpatient rehabilitation had lower odds of independence after 1 year and 5 years. Notably, these participants had more severe stroke and more comorbidities, and were more often diagnosed with haemorrhagic stroke. Compared with those with ischaemic stroke, people with haemorrhagic stroke reportedly have a lower functional status after 1 year (20). Another previous study classified individuals with first-time ischaemic stroke diagnoses, according to stroke severity, functional dependence, and comorbidities, and reported that the group with the most severely affected participants exhibited a late gain-of-function after over 1 year (21). This is relevant when interpreting the results from our study. In current clinical practice, rehabilitation is focused on the first year after stroke, in accordance with national guidelines (22) and current evidence (23). This seems to be suitable for participants who can participate in home-based or outpatient rehabilitation. Our present results demonstrated that among participants who were independent at 1 year – regardless of whether they had planned rehabilitation – there was a high probability of remaining independent at 5 years. Furthermore, participants who were dependent at 1 year were less likely to become independent after 5 years. This is in line with previous findings that most clinical improvement occurs during the first year after stroke (23). On the other hand, we suspect that 1 year of rehabilitation is insufficient for patients with severe stroke and other complicating factors. The implementation of a second evaluation, followed by an individualized rehabilitation period, might potentiate improved function for patients with low initial functional capacity. Evidence indicates that multimodal rehabilitation performed later than 1 year after stroke can improve functioning (24), and it may be helpful to assign rehabilitation differently for patients with mild and moderate stroke, so-called slow-stream rehabilitation (25), which is presently lacking in Sweden.

Regarding the participants without planned rehabilitation, clinical polarization could be suspected. This group comprised individuals who were either discharged with minimal impairments, or deemed unlikely to benefit from standard inpatient rehabilitation due to factors such as advanced age, comorbidities, or cognitive impairment. We found that participants in this group were largely either independent or deceased after 5 years, with fewer being dependent. The present findings emphasize the importance of monitoring not only medical aspects but also long-term rehabilitation needs, and providing rehabilitation adapted for people with severe stroke (25). To address this gap, guidelines recommend that patients who initially appear unfit for rehabilitation upon discharge from a stroke unit care should be reassessed at regular intervals. This recommendation is a promising step towards enhancing these patients’ prospects for functional recovery, inviting speculation as to its potential impact on the prognosis of stroke patients who are initially unable to participate in standard inpatient rehabilitation (26, 27).

In the present study, rehabilitation was categorized into 3 pathways, each of which incorporates a broad variation of rehabilitation forms. In primary care, rehabilitation is often more generalized, focusing on a broad range of needs. On the other hand, specialized clinics can offer more intensive targeted rehabilitation, with state-of-the-art therapies that are tailored to specific disabilities or conditions. This contrast is even more pronounced when comparing basic home-based rehabilitation to early supported discharge programmes (3). Home-based rehabilitation typically involves less frequent therapist visits and a reliance on self-managed exercises, whereas early supported discharge programmes are more comprehensive and involve a multidisciplinary team approach to support a smoother transition from hospital to home (2, 3). Hence, when lumping these diverse approaches under the broad categories of outpatient rehabilitation and home-based rehabilitation, there is a risk of overlooking the nuances and specific benefits of each approach. This oversimplification may lead to misconceptions concerning efficacy, and could influence the results and interpretations of studies comparing different rehabilitation modalities.

### Strength and limitations

The main strengths of this study are its longitudinal design, long-term follow-up, and national coverage with participation from all socioeconomic groups. The large national coverage implies that our results can be generalized to other samples with a similar distribution and organization of rehabilitation. The ordinal logistic regression models were developed using a training set and then evaluated using separate data, which enabled prediction of a dependent variable. The survey questions used to assess functional outcomes were previously transformed into the modified Rankin Scale with high precision (28, 29). This supports the adequate validity of our outcome categories.

This study had a few limitations. Our aim was to investigate the relationship between functional outcomes and planned rehabilitation after discharge from stroke units. However, it remains unknown whether the planned rehabilitation was realized or re-evaluated during the rehabilitation period. Additionally, stroke severity was assessed using the proxy of consciousness on admission, which was assessed using the RLS-85 (6). The level of consciousness is considered a reliable predictor of post-stroke survival (5, 6). Another limitation is the absence of data regarding functional status on discharge, which necessitates cautious interpretation of the transition probabilities. Compared with the excluded individuals, the participants included in our sensitivity analyses had a lower median age and a higher proportion of being alert on hospital admission. This was expected, as independence before stroke was an inclusion criterion.

### Conclusion

Achieving independence at 1 and 5 years after a stroke was more likely with planned rehabilitation in a home setting or outpatient care. Conversely, those designated for inpatient rehabilitation had reduced chances of achieving independence, a finding that aligns with expectations. Adherence to current stroke rehabilitation guidelines appears to effectively support recovery for those undergoing home-based or outpatient rehabilitation. Patients assigned to inpatient rehabilitation, who are often facing more complex health challenges, may benefit from a prolonged rehabilitation process that includes regular reassessments or a gradual sustained approach to care.

## Supplementary Material

PREDICTION OF LONG-TERM FUNCTIONAL OUTCOME FOLLOWING DIFFERENT REHABILITATION PATHWAYS AFTER STROKE UNIT DISCHARGE
