# Supplementary material for: PREDICTION OF LONG-TERM FUNCTIONAL OUTCOME FOLLOWING DIFFERENT REHABILITATION PATHWAYS AFTER STROKE UNIT DISCHARGE
Source: J Rehabil Med. 2024 May 21;56:19458. doi: 10.2340/jrm.v56.19458 (PMC11135335; doi:10.2340/jrm.v56.19458)
Supplement: PREDICTION OF LONG-TERM FUNCTIONAL OUTCOME FOLLOWING DIFFERENT REHABILITATION PATHWAYS AFTER STROKE UNIT DISCHARGE [file JRM-56-19458-s1.pdf]

Supplementary material has been published as submitted. It has not been copyedited, or typeset by Journal of Rehabilitation Medicine

Table SI. Classification table for the training set at the 1-year follow-up (*n* = 4018)

	**Predicted; Independent**	**Predicted; Dependent**	**Predicted; Dead**
**Independent**	2305	57	101
**Dependent**	517	68	180
**Dead**	440	56	294

**Correct predictions:**	2667
**Incorrect predictions:**	1351

Table SII. Classification table for the test set at the 1-year follow-up (n = 1004)

	**Predicted; Independent**	**Predicted; Dependent**	**Predicted; Dead**
**Independent**	574	10	26
**Dependent**	133	19	44
**Dead**	111	9	78

**Correct predictions:**	671
**Incorrect predictions:**	333

Table SIII. Classification table for the training set at the 5-year follow-up ($n = 4018$)

	**Predicted; Independent**	**Predicted; Dependent**	**Predicted; Dead**
**Independent**	1283		418
**Dependent**	180		237
**Dead**	390		1510

**Correct predictions:**	2793
**Incorrect predictions:**	1225

Table SIV. Classification table for the test set at the 5-year follow-up (*n* = 1004)

	**Predicted; Independent**	**Predicted; Dependent**	**Predicted; Dead**
**Independent**	309		107
**Dependent**	54		51
**Dead**	84		399

**Correct predictions:**	708
**Incorrect predictions:**	296
